# Effect of Weed Management on the Parasitoid Community in Mediterranean Vineyards

**DOI:** 10.3390/biology10010007

**Published:** 2020-12-24

**Authors:** Gabriella Möller, Tamar Keasar, Idan Shapira, Daniella Möller, Marco Ferrante, Michal Segoli

**Affiliations:** 1 Mitrani Department of Desert Ecology, The Jacob Blaustein Institutes for Desert Research, Ben-Gurion University of the Negev, Sede Boqer Campus, Sede Boqer 8499000, Israel; gabriellamoller1@gmail.com (G.M.); moller.daniella@gmail.com (D.M.); msegoli@bgu.ac.il (M.S.); 2 Department of Biology, University of Haifa—Oranim, Tivon 36006, Israel; 3 Department of Evolutionary and Environmental Biology, University of Haifa, Haifa 3498838, Israel; shapiraidan@gmail.com; 4 Megiddo Biosphere Reserve, Megiddo Regional Council, Megiddo 1893300, Israel; 5 Azorean Biodiversity Group, CE3C—Centre for Ecology, Evolution and Environmental Changes, Faculty of Agricultural and Environmental Sciences, University of the Azores, PT-9700-042 Angra do Heroísmo, Portugal; marco.ferrante@live.it

**Keywords:** conservation biological control, community composition, natural enemies, non-crop vegetation, parasitoids, vineyards, weed management

## Abstract

**Simple Summary:**

Parasitoid wasps control insect pests in agricultural crops, but often require additional resources from non-crop plants. Vineyard growers sometimes address this need by planting or sowing pre-selected herbs around the plots or between the vine rows. Here, we explored the simpler strategy of conserving spontaneously growing weeds within Mediterranean vineyards, and trimming them mechanically when they reach large size and interfere with farming activities. We compared this strategy with matched plots, in which resident weeds were sprayed regularly with herbicides, representing the conventional treatment. As predicted, overall parasitoid abundance and the number of parasitoid species were higher in the weed conservation plots. However, the direction and magnitude of the effect differed between the dominant parasitoid species, and populations of some potential pests increased in the weed conservation treatment. Conservation of weeds that grow spontaneously in vineyards is a low-cost practice that offers multiple benefits, such as reduced soil erosion, stabilization of soil temperatures, and diminished exposure of farmers to agrochemicals. Our results show that communities of important biological control agents may gain from this practice as well. Nevertheless, weed conservation within vineyards can only be sustainable if its benefits outweigh the risks of attracting crop pests.

**Abstract:**

Enriching agroecosystems with non-crop vegetation is a popular strategy for conservation biocontrol. In vineyards, the effects of specific seeded or planted cover crops on natural enemies are well-studied, whereas conserving spontaneously developing weeds received less attention. We compared parasitoid communities between matched pairs of vineyard plots in northern Israel, differing in weed management practices: “herbicide”, repeated herbicide applications vs. “ground cover”, maintaining resident weeds and trimming them when needed. Using suction sampling, we assessed the parasitoids’ abundance, richness, and composition during three grape-growing seasons. Ground cover plots had greater parasitoid abundances and cumulative species richness than herbicide-treated plots, possibly because of their higher vegetation cover and richness. Dominant parasitoid species varied in their magnitude and direction of response to weed management. Their responses seem to combine tracking of host distributions with attraction to additional vegetation-provided resources. Parasitoid community composition was mildly yet significantly influenced by weed management, while season, year, and habitat (weeds vs. vine) had stronger effects. Vineyard weeds thus support local biocontrol agents and provide additional previously demonstrated benefits (e.g., soil conservation, lower agrochemical exposure) but might also attract some crop pests. When the benefits outweigh this risk, weed conservation seems a promising step towards more sustainable agricultural management.

## 1. Introduction

Conservation biological control (CBC) practices aim at enhancing the resident natural enemies of agricultural pests via manipulations of the local environment [1,2]. One CBC strategy is to increase or preserve the non-crop vegetation that grows naturally within or near an agricultural field [3]. Maintaining vegetation cover may provide benefits, such as preventing soil erosion, nutrient enrichment of the soil, water quality control, and biodiversity enhancement and conservation [4,5]. Moreover, non-crop vegetation may provide refuge and food resources for natural enemies, increasing their abundance, richness, and effectiveness for pest control [6,7]. A rich and diverse community of natural enemies may contain potential biological control agents that are adapted to varying conditions and microhabitats and attack a wide range of crop pests [8,9,10,11]. Thus, preserving non-crop vegetation could potentially complement or replace pesticide applications in crop fields while benefiting local biodiversity and the environment [4,6].

The effect of non-crop vegetation, adjacent to or inside the crop field, on natural enemies has been studied in a variety of cropping systems, including peach [12], apple [13,14] and olive [15] orchards, cucumber [16], wheat [17], cabbage [18], cauliflower, soybean, eggplant, and potato fields (reviewed in [19]). Some of these manipulations resulted in successful pest control [13,16,18,19], while others had no effect [20], or even showed negative effects on pest control [12,14,15,21]. These mixed results raise the need for crop- and site-specific investigations to guide sustainable agricultural practices.

Vineyards are intensively managed monocultures with little native vegetation within the crop plots, as weeds are typically controlled using herbicides. However, there is evidence that non-crop vegetation in vineyards may increase the biodiversity of many plant and animal taxa and improve the delivery of ecosystem services [22]. For example, in Californian vineyards, non-crop vegetation has been found to enhance the control of leafhoppers and thrips by providing refuge for their associated natural enemies [23]. Similar findings suggesting benefits of non-crop vegetation for natural enemies have been reported for vineyards in Australia [24,25], New York State [26], New Zealand [4], Italy [27], and Israel [28]. However, these and most other studies investigating the effects of non-crop vegetation on natural enemies within vineyards tested the value of potted, planted, or sown plants, rather than the effect of naturally growing vegetation. These studies normally focused either on one or on a mixture of a few selected species, often irrespective of whether they are native or exotic [4].

In comparison to exotic plants, naturally growing vegetation is more likely to attract native natural enemies and to be better adapted to local conditions [29]. For example, naturally growing ground cover vegetation increased parasitism rates on leafhoppers and decreased their crop infestations in vineyards in California [29], and increased the abundance of beneficial soil entomopathogenic nematodes in Spanish vineyards [30]. Additionally, spontaneously developing vegetation may require fewer resources and labor (e.g., sowing, repeated planting) than supplemented vegetation [6]. Unlike sown cover crops, naturally growing plants do not involve the risk of poor establishment [31]. Importantly, native regional plants, which often dominate naturally developing plant communities in agroecosystems, are also more likely to promote biodiversity conservation [4]. Nonetheless, studies assessing the impact of spontaneously developing non-crop vegetation on natural enemies in vineyards are scarce.

We manipulated the weed management practice and assessed the resulting abundance and diversity of natural enemies in wine-producing vineyards in northern Israel. Our experimental plots were either completely treated with herbicides (herbicide treatment), or treated with herbicides under the grapevine rows only, while allowing the spontaneous growth of natural vegetation between the rows (ground cover treatment). We focused on hymenopteran parasitoids—a highly diverse group that includes many effective biological control agents [32,33]. Considering the resources and habitat potentially provided by the non-crop vegetation [6], we predicted a higher abundance and diversity of parasitoid wasps in ground cover plots than in herbicide plots throughout the grape-growing season. We further hypothesized that the response to weed management would differ among parasitoid species, due to their diverse habitat preferences and resource requirements, leading to changes in parasitoid community composition between treatments. This would be in accordance with previous data from pomegranate orchards in Israel [34,35]. To our knowledge, this is the first experimental test of the effects of herbicide use on parasitoid communities in vineyards.

## 2. Materials and Methods

*Study sites:* From 2016 to 2018, we sampled six wine-producing vineyards located in the Mediterranean climate area of northern Israel (Supplementary Materials, Table S1). Four vineyards were located in a hill-surrounded valley, at an altitude of 350–450 MASL, with soils composed of a mixture of volcanic tuff, gravel, and terra rossa. Winter temperatures range from 4 to 16 °C, summer temperatures range from 15 to 29 °C, and the annual average precipitation is 671 mm in this region. The two remaining vineyards were located on a volcanic plateau at 900 MASL, with the soil composed of volcanic tuff and basalt. The ranges of winter and summer temperatures in this region are 0–15 °C and 12–30 °C, respectively, and the annual precipitation fluctuates between 800 and 1000 mm. In these vineyards, the bud break (start of leaf growth from buds) begins in early to mid-March. Flowering occurs in mid-April, and fruits start growing around two weeks later. Grape harvest occurs from August to September. The natural vegetation is mainly composed of annuals that flower in the spring (March–May) (Supplementary Materials, Table S2). The main insect pests attacking the vineyards are the European grapevine moth (*Lobesia botrana*: Lepidoptera), the citrus mealybug (*Planococcus citri*: Hemiptera), and a few leafhopper species of the genera *Empoasca* and *Zygina* (Hemiptera). We refer to these species collectively as “leafhoppers”, as it is extremely difficult to distinguish between them morphologically (Rakefet Sharon, pers. comm.).

*Weed management treatments:* In each vineyard, two plots (4–10 ha) with identical grape varieties (Supplementary Materials, Table S1) and agronomical regimes were selected to compare the effects of two weed management practices on the parasitoid communities. Insecticides (such as imidacloprid, cypermethryn, thiamethoxam, emamectin benzoate, lufenuron, flonicamid, and spirotetramat) were applied mainly to control the European grapevine moth and aphids. The specific insecticide products and spray dates varied between vineyards, but paired plots received similar treatments (for details see pesticide applications, Table S3). Due to logistical constraints, we had to stop sampling some of the vineyards after the first or the second field season, and replaced them with other vineyards for the remaining study period (Table S1). These changes did not disrupt our study, however, since we used a paired experimental design. Namely, in one plot of each vineyard, herbicide was used under the grapevine rows only, while the naturally growing vegetation between the vine rows was retained until the end of the herb flowering season, and then mechanically trimmed (hereinafter “ground cover”). The other plot was treated with herbicides throughout, to prevent herbaceous vegetation from growing (hereinafter “herbicide”). Herbicide spray was mostly applied before herb germination in winter.

*Vegetation cover and richness*: For all plots, vegetation cover (green plant material only) and the identity of plant species in the non-crop area between the grapevines were recorded along three 25 m transects per plot at the same periods of arthropods sampling. The dominant plant species in each transect were noted. Vegetation coverage was estimated visually, using a 1–5 scale representing: very low (<20%), low (20–40%), medium (40–60%), high (60–80%), and very high (>80%) cover. All vegetation surveys were conducted by a single trained observer to reduce estimation bias in vegetation cover scores.

*Arthropod collection:* Arthropods were collected using a Vortis suction sampler (Burkard Manufacturing Co. Ltd., Rickmansworth, UK), vacuuming the vegetation for 15 s per sample with a distance of at least 5 m between samples and avoiding the plot edges. For each plot there were four sampling points, and at each point we sampled both the area between the vines where the non-crop vegetation naturally grows (hereinafter referred to as “grass” habitat), and the vine foliage (“vine” habitat). Samples in the grass habitat were taken from the vegetation between the vine rows, even if it was scarce, rather than from the bare ground, which is not a suitable habitat for parasitoids. Each plot was sampled three times during the grape-growing season until a few weeks before harvest (see Supplementary Materials, Table S1 for sampling dates).

Samples were preserved in 70% ethanol at 4 °C. All arthropods were identified at least to order level. Parasitoid wasps were identified to the genus or species level using keys [36,37,38,39,40] and consulting experts. When species/genus identification was not possible, wasps were identified to family and sorted into morphospecies.

*Data analyses*: All statistical analyses were carried out using the R software version 3.5.1 [41] using the packages *glmmTMB* [42], *lme4* [43], *pairwiseAdonis2* [44], *plotrix* [45], *vcd* [46], *vegan* [47], and *VGAM* [48].

*Vegetation cover and richness*: We evaluated the effects of treatment and season on vegetation cover (a discrete variable with five levels) and plant species richness. We used an ordinal logistic regression for the vegetation cover analysis. A Generalized Linear Mixed Model (GLMM) with a Poisson link function and vineyard as a random-intercept factor was used for the species richness analysis.

*Parasitoid abundances:* Total parasitoid counts from the four samples of each plot-habitat combination were averaged, rounded to whole numbers, and inspected for zero-inflation and over-dispersion. GLMMs with negative binomial link functions were used to test for factors affecting the abundances of all parasitoids, and of each of the five most dominant parasitoid morphospecies (which constituted ≥5% of total parasitoid abundance and occurred in at least five of the six vineyards). The fixed factors included in the initial models were weed management (herbicide/ground cover), season (leaf growth/fruit growth/ripening), year (2016–2018), habitat (vine/grass), and all the two-way interactions between management and the other factors. Sampling year was considered as a fixed effect, rather than random, because of its small number of levels [49]. The vineyard ID was included as a random-intercept factor to account for the paired experimental design and for the repeated sampling in each vineyard [50].

Information about the identity of potential arthropod hosts for each of the dominant parasitoid species was compiled from taxonomic keys and published literature. The abundance of the potential arthropod hosts was also included as a covariate in the models for the individual dominant morphospecies, except for *Telenomus* sp. This is because some of its putative hosts (Lepidoptera) were under-represented in our suction samples, and other potential hosts (Heteroptera) were not sufficiently identified.

Likelihood ratio (LR) tests, which compared increasingly reduced models, were used to determine the best fitting model [51].

*Leafhopper and thrips abundances:* Leafhoppers are potential hosts of three dominant parasitoid morphospecies in our samples: *Lymaenon litoralis*, *Anagrus* sp., and *Oligosita* sp., whereas thrips are potential hosts of *Ceranisus* sp. To test the effects of the vegetation management treatment directly on these herbivores, we ran two additional GLMMs, treating leafhopper and thrips abundances as their respective dependent variables. Explanatory factors were defined as in the parasitoid abundance models.

*Parasitoid species richness:* We compared species accumulation curves for the parasitoids across vegetation treatments and habitats. To estimate the associated confidence intervals, we resampled the data at a random order 100 times, without replacement.

Community composition analyses: Permutational Multivariate Analysis of Variance (PERMANOVA) tests were used to examine differences in the composition of parasitoid species assemblages [47,52]. The influence of the different covariates (herbicide management, sampling season, and all two-way interactions with management) on the species assemblages was evaluated. Using the full parasitoid community violated the assumption of homogeneity in dispersion within groups. We therefore report on a PERMANOVA analysis that considers only the five dominant morphospecies. Using the full dataset did not change the results, but lowered the explained variance. PERMANOVA tests were based on Bray–Curtis distance matrices and run for 999 permutations. Data were stratified at the vineyard level to account for possible differences between vineyards.

## 3. Results

### 3.1. Vegetation Cover and Richness

**Figure 1:**
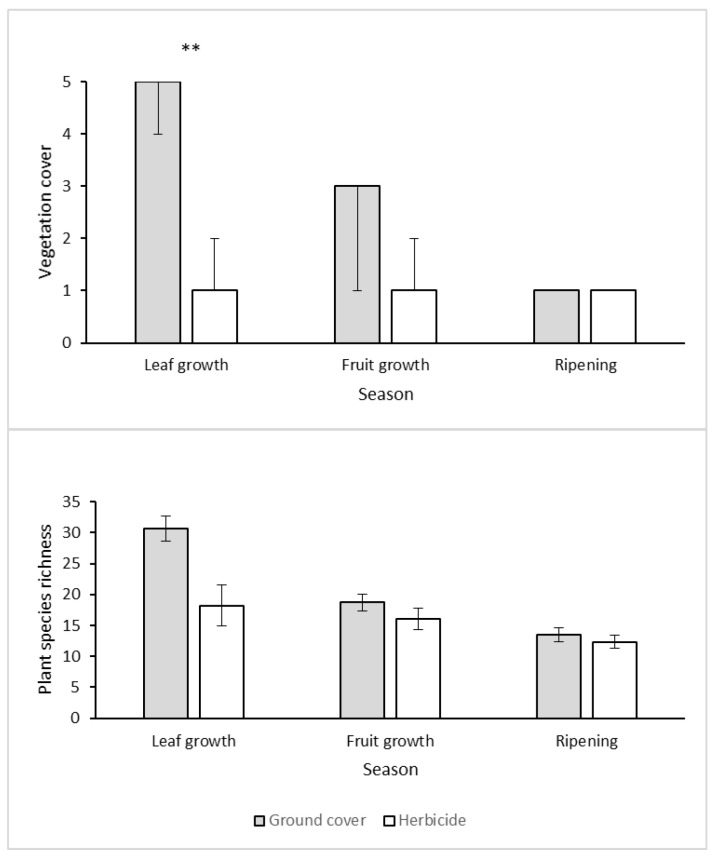
Top: Median vegetation cover scores (on a 1–5 scale) with their associated interquartile intervals, grouped by weed management treatment and season. Note that the 75% quartile line overlaps with the median in the ground cover treatment, while the 25% quartile line overlaps with the median in the herbicide treatment. Bottom: Mean ± SE plant species richness. ** Significant effect of treatment (*p* < 0.01) in a post-hoc test.

Vegetation cover decreased from vine leaf-growth (spring) to fruit ripening (mid-summer) in the ground cover treatment, and remained consistently low in the herbicide treatment. Plant species richness decreased from vine leaf growth to fruit ripening in both treatments and in all three years, and was consistently higher in the ground cover plots than in the herbicide plots (Figure 1). Accordingly, vegetation cover was significantly influenced by both treatment (*p* = 0.046) and by its interaction with season (*p* = 0.003, due to the difference between the leaf-growth and fruit-growth seasons, ordinal logistic regression). Plant species richness was affected by treatment, season, and their interaction (GLMM, *p* < 0.001 for all effects). Vegetation cover and richness were highly correlated (Spearman’s rank correlation: rho = 0.63, *p* < 0.001). We concluded that the herbicide treatment was successful in reducing plant cover and richness as compared to the ground cover treatment, especially during leaf growth in the spring.

### 3.2. Collected Arthropods and Parasitoids

From 2016 to 2018, 35,277 arthropods were collected. The most abundant insect orders were Thysanoptera (32.7% of the total captured), Hemiptera (22.5%) and Hymenoptera (15.4%), the majority of which were parasitoid wasps (12.6% of all arthropods, *n* = 4455). Other non-insect arthropods sampled in the vineyard belonged to Acari (9.4%) and Araneae (2.8%). The majority of arthropods (83.3%) and parasitoids (73.4%) were captured in the grass habitat rather than in the vine foliage.

### 3.3. Parasitoid Abundances

**Figure 2:**
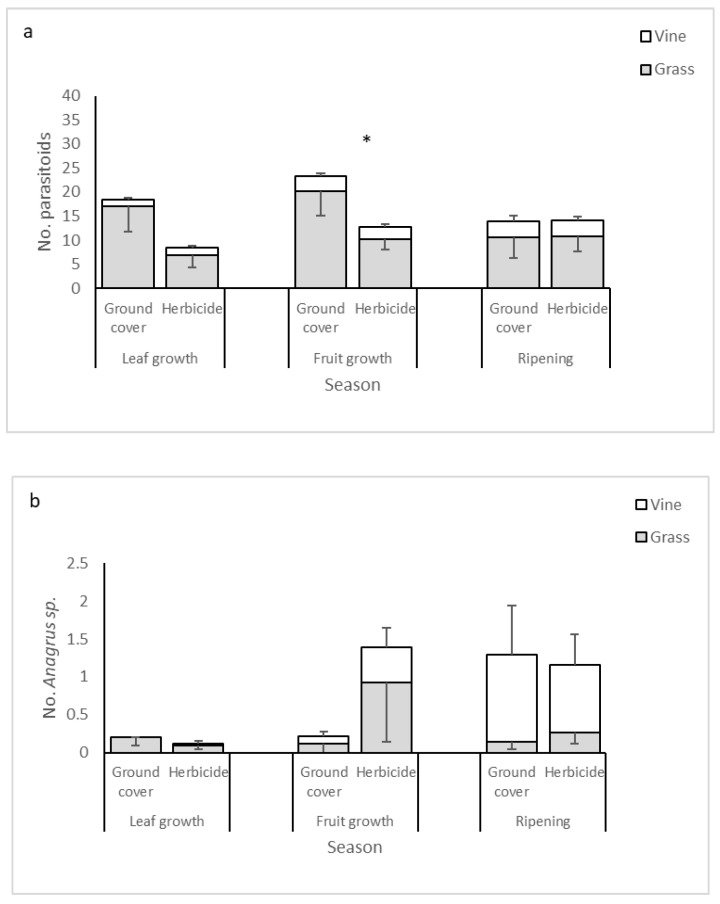

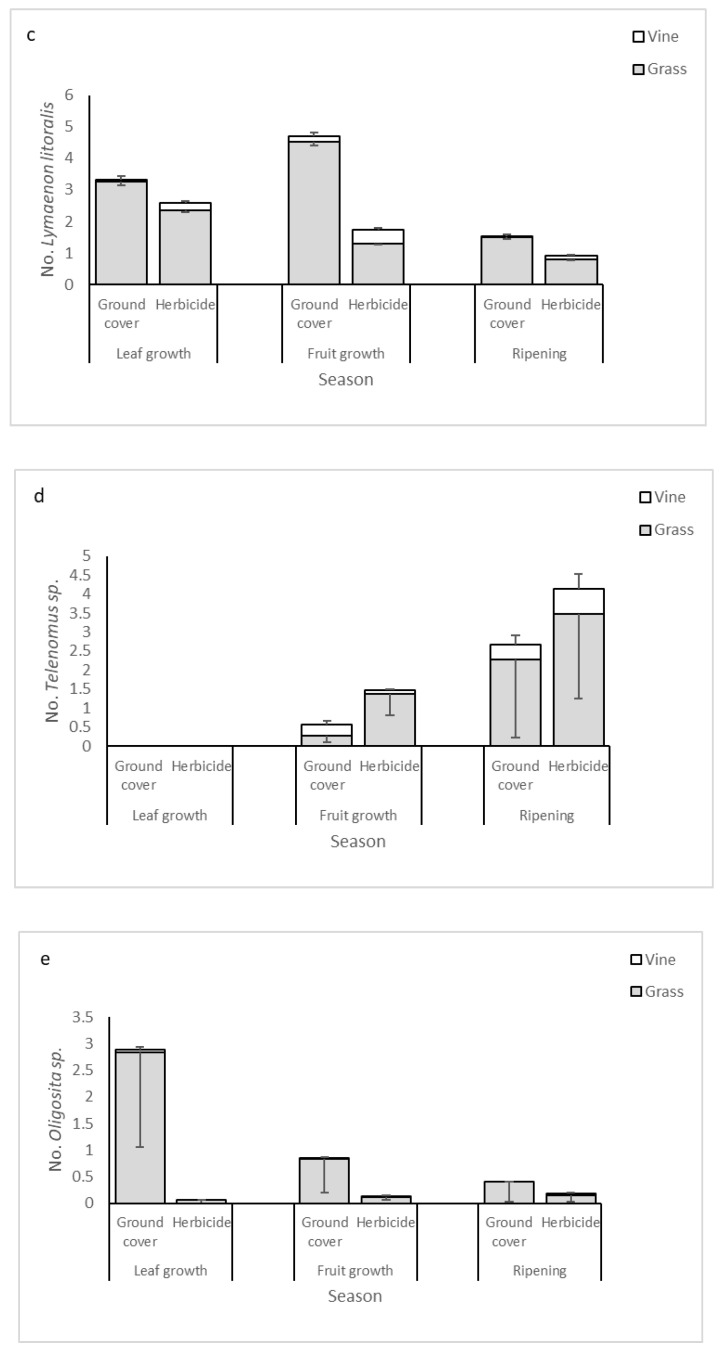

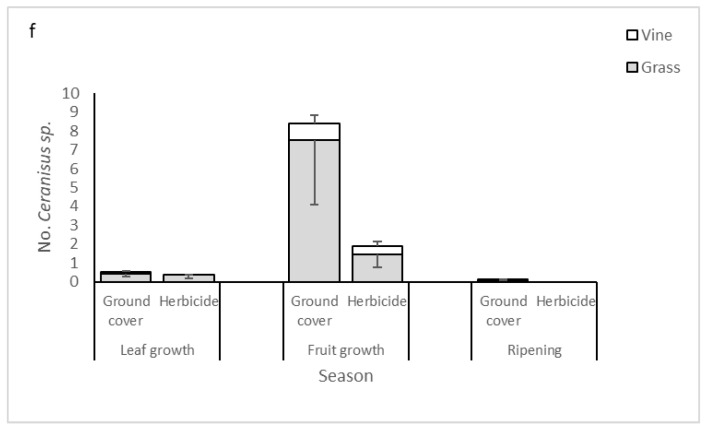
Mean ± SE abundance of all parasitoids combined (**a**) and of the five dominant species in the suction samples from the grass (grey bars) and vine (white bars) habitats. (**b**) *Anagrus* sp.; (**c**) *Lymaenon litoralis*; (**d**) *Telenomus* sp.; (**e**) *Oligosita* sp.; (**f**) *Ceranisus* sp. * denotes a significant difference between treatments in Tukey’s post-hoc test (*p* < 0.05).

Parasitoid mean abundances were higher in the ground cover than in the herbicide plots. They were also higher in the grass habitat than in the vine foliage. In addition, abundances were affected by sampling year, being higher in 2018 than in the former years. (Table 1, Figure 2a).

### 3.4. Dominant Parasitoid Species

Five dominant species/morphospecies constituted 49% of the parasitoids captured in the vineyards. They were *Lymaenon litoralis* (Haliday, 1833) (Family Mymaridae) (16% of all parasitoid individuals), *Ceranisus* sp. (Eulophidae) (13%), *Telenomus* sp. (Platygastridae) (10%), *Anagrus* sp. (Mymaridae) (5%), and *Oligosita* sp. (Trichogrammatidae) (5%).

The most abundant parasitoid, *L. litoralis*, was consistently and significantly more abundant in the ground cover plots than in the herbicide plots. *Ceranisus* sp. and *Oligosita* sp. also trended towards higher abundance in the ground cover plots, while *Telenomus* sp. showed the opposite trend (Table 1, Figure 2b–f). The dominant parasitoid species exhibited variable seasonal patterns. For example, *L. litoralis* and *Oligosita* sp. were more abundant at the beginning of the grape-growing season, while *Anagrus* sp. and *Telenomus* sp. increased in abundance towards the end of the season (Figure 2). Further, the sampling year (as a main factor or in interaction with weed management) had significant or near-significant effects on *Anagrus* sp., *Oligosita* sp., and *Telenomus* sp. (Table 1). Most dominant species were more abundant in the grass habitat than in the vine habitat, but *Anagrus* sp. did not show a clear habitat preference (Figure 2b). For the four species in which host effect could be evaluated, parasitoid abundance was positively related to the abundance of their potential hosts (Table 1).

### 3.5. Main Groups of Potential Hosts

**Figure 3:**
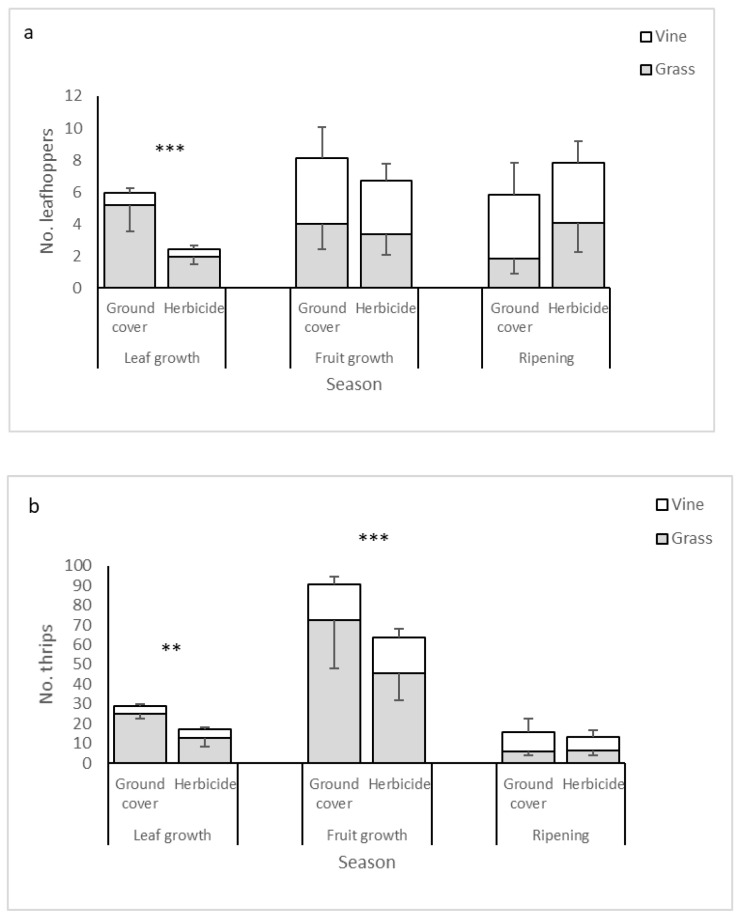
Mean ±SE abundance of leafhoppers (**a**) and thrips (**b**) in the suction samples from the grass (grey bars) and vine (white bars) habitats. Asterisks denote significant differences between treatments in Tukey’s post-hoc tests (** *p* < 0.01, *** *p* < 0.001).

Leafhoppers, potential hosts of *L. litoralis*, *Oligosita* sp., and *Anagrus* sp., were more abundant in the ground cover plots than in the herbicide plots, especially during leaf growth. They occupied both the vine and the grass habitats (Table 1, Figure 3a). Thrips, potential hosts of *Ceranisus* sp., were also more abundant in the ground cover treatment than in the herbicide treatment, peaked during fruit growth and were sampled mainly in the grass habitat (Table 1, Figure 3b).

### 3.6. Parasitoid Species Richness

**Figure 4:**
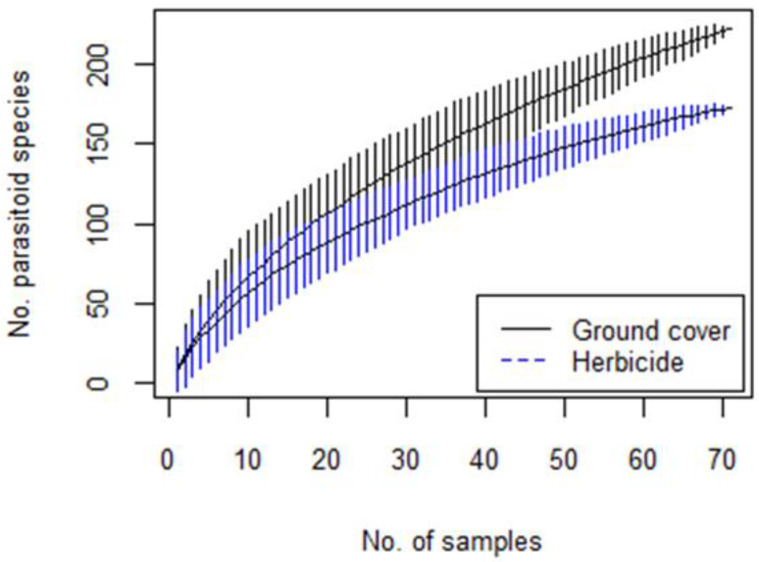
The cumulative number of parasitoid species in the ground cover and herbicide plots, with associated 95% confidence intervals.

Of the 279 parasitoid morphospecies identified in the vineyards, 113 were singletons (~41% of all morphospecies) and 43 were doubletons (~15% of all morphospecies). The cumulative richness of parasitoid species was consistently higher in the ground cover plots than in the herbicide plots. This difference was statistically significant when the complete species assemblage (pooled over all plots, seasons, and years) was considered (Figure 4). In addition, the species accumulation curve was significantly higher in the grass habitat than in the vines (Supplementary Materials, Figure S1).

### 3.7. Community Composition

PERMANOVA results indicate that the composition of the dominant parasitoid species was mostly affected by the sampling season, year, and habitat (Table 2). Although weed management and its interaction with habitat significantly affected the species composition, each of these factors explained only a small proportion (<2%) of the variance. The effect of the year needs to be cautiously interpreted since the data violated the assumption of homogeneity of dispersion within groups.

We used 50 species/morphospecies, each of which was represented by >10 individuals in our dataset, to estimate habitat specialization at the parasitoid community level. Forty-five of those species were sampled both in the vine and in the grass habitats, five were only sampled in the grass, and none were sampled in the vines only. Hence, most parasitoids were found in both habitats, albeit to different degrees.

## 4. Discussion

We examined the effects of weed management practices on parasitoid communities in vineyards in Israel. We found that the ground cover plots supported higher overall parasitoid richness and abundance than the herbicide-treated plots, in particular during leaf growth and fruit ripening. This result can be explained by the higher vegetation cover and richness in the ground cover plots, which was most pronounced early in the season. Notably, weed management effects might have been underestimated in this study as the sampling effort was standardized by vacuuming the vegetation for a fixed duration, rather than by sampling a fixed area within the vineyard. Given the consistently higher vegetation cover in the ground cover plots than in herbicide plots, the differences in total per-vineyard abundance and richness of parasitoids are expected to be even higher.

Our results are consistent with previous studies that explored the effects of non-crop vegetation on natural enemies in agro-ecosystems, suggesting that the vegetation may provide direct benefits, such as sugar resources, shelter, and favorable microclimatic conditions for the parasitoids [3,6]. However, herbaceous vegetation in the vineyards may also provide additional food resources to herbivorous insects, allowing their populations to increase (e.g., [12,14,15,21]). This can, in turn, indirectly support larger numbers of parasitoids. Indeed, both thrips and leafhoppers were found to be more abundant in the ground cover plots, at least during part of the season. Our statistical modeling allowed us to separate the direct effects of the vegetation management treatment on parasitoid abundance from the indirect effects mediated by their host abundance, for four dominant species. All four species (*Anagrus* sp., *L. litoralis*, *Oligosita* sp. and *Ceranisus* sp.) were affected by the abundance of their potential host group. However, *L. litoralis* was significantly and positively affected by the ground cover vegetation treatment as well, even after statistically controlling for the effect of its host (leafhoppers). In *Oligosita* sp. and *Ceranisus* sp. (parasitizing leafhoppers and thrips, respectively), a similar trend was observed, but it was only marginally significant. Thus, the ground cover treatment probably benefited parasitoids both by attracting their hosts and by providing them with additional non-host resources.

Interestingly, the maintenance of ground cover negatively affected *Telenomus* wasps, and had an inconsistent effect on *Anagrus* sp. Likely, these effects were mediated through changes in the vegetation, and particularly in the density of specific plants that may be important as sources of food or hosts for the parasitoids. For instance, plants of the genus *Chenopodium*, which include herbicide-resistant weeds and were dominant in our herbicide-treated plots (Supplementary Materials, Table S2), were previously reported to attract *Telenomus* species [53]. Similarly, in pomegranate orchards, another widespread crop in Mediterranean regions, parasitoid abundance showed species-specific responses to plant-related variables [34,35].

Dominant species also varied in their seasonal patterns, with *L. litoralis* occurring throughout the season, *Oligosita* sp. peaking early in the season, *Ceranisus* sp. mostly in mid-season, and the other two species being mostly abundant late in the season. This may suggest their potentially complementary function in regulating pest populations throughout the season. Such seasonal complementarity between parasitoids and ground-dwelling predators was shown to improve the biological control of pollen beetles in oilseed rape fields [54].

Total parasitoid abundance, as well as the abundance of several of the dominant species, also showed significant or near significant variation between years. Similarly, yearly fluctuations in population densities have been reported for *Anagrus* parasitoids in temperate vineyards [26]. High inter-annual variations in population densities were also described for hymenopteran pollinators in Mediterranean ecosystems [55,56]. Taken together, these fluctuations highlight the need for large-scale, long-term monitoring when evaluating anthropogenic effects on insect communities. Our three-year study could provide a valuable starting point for such a monitoring project.

Finally, with the exception of *Anagrus* sp. (leafhopper egg parasitoid), parasitoid abundance was higher in the grass compared to the vine habitat. This is consistent with previous studies of parasitoids in Israeli vineyards [57,58]. Importantly, although fewer parasitoids were captured in the vine foliage, in most cases we found no significant interaction between the herbicide treatment and the habitat in affecting parasitoid abundance (Table 1), suggesting that the treatment had a similar effect in both habitats. The lower capture rate in the vines may partially reflect a sampling artifact, as the vine foliage is more complex and three-dimensional, while the ground vegetation is more easily accessible for suction sampling. Another possibility is that parasitoids reached both habitats but spent more time in the grassy vegetation as it provides them with additional resources. Some parasitoids indeed never visited the vines, suggesting no or low biological control services provided by these species. This is also reflected by the significant treatment × habitat effect on parasitoid community composition (Table 2). However, most species (45 out of 50 with more than 10 captured individuals) were found in both habitats. We conclude that most parasitoids used both habitats, although probably to different extents.

In addition to the effect of weed management on parasitoid abundances, the species accumulation curves indicated higher parasitoid species richness in the ground cover plots than in the herbicide plots. This is consistent with previous studies showing an increase in natural enemy diversity in complex environments with high vegetation cover and richness [11], and specifically with the results of experimental manipulations of non-crop vegetation in vineyards [22]. A rich parasitoid community may enhance the control of different pests under varying environmental conditions [9,11].

As predicted, weed management also significantly influenced parasitoid community composition. However, the treatment effect explained less than 2% of the variance, which instead depended mostly on the sampling season, year, and habitat. Indeed, temporal dynamics related to the availability of resources (e.g., flowers or prey populations) and changes in habitat quality have been found to be important in determining arthropod densities and habitat preference [8,59]. Note that our analysis is stratified by vineyard, and hence, it does not reflect the effect of geographical location on community composition.

In Israeli viticulture, the traditional weed management approach has been to maintain “clean vineyards” by regularly applying pre-emergent herbicides in the fall and in the spring. Health concerns, the rising costs of herbicides, and the increases in herbicide-resistant weeds motivate farmers to shift to the conservation of spontaneous ground cover [60]. Based on our study, we predict some potentially beneficial consequences of this management change at least for some parasitoid species in the vineyards, although these benefits should be weighed against potential increases in the pest populations. Conservation of non-crop vegetation provides additional benefits, such as reduced soil erosion [61], improved soil quality [62,63], grapevine growth, and grape quality [64]. Compared to the supplementation of non-natural vegetation, the maintenance of spontaneous vegetation has the potential to be more easily accepted by the farmers and integrated into the management practices. Indeed, the farmers involved in this study who gained experience with the ground cover practice showed a positive attitude towards it [28].

## 5. Conclusions

Agricultural areas occupy about 40% of the ice-free surface of our planet [65], and the conventional agricultural system has a considerable impact on biodiversity and the associated ecosystem services [66,67]. Sustainable alternatives, such as the ones proposed in the CBC framework, can help to mitigate the ongoing decline in arthropod diversity and biomass [68,69]. Studies such as ours provide the information base for stakeholders to simultaneously maintain crop yields while preserving rich and healthy communities of beneficial arthropods in cultivated areas.

## Figures and Tables

**Table 1:** Results from Linear Mixed Models showing the effect of explanatory variables on the abundances of all parasitoids, each of the five dominant parasitoid species/morphospecies, and two groups of potential hosts. Variables selected by maximum likelihood ratio tests with *p* < 0.1 are shown: df, degrees of freedom; LRT, likelihood ratio test statistic.

Explanatory Variables	df	LRT	*p*
Parasitoids			
Management	1	4.04	0.044
Habitat	1	36.23	<0.001
Year	2	7.43	0.024
***Lymaenon litoralis***			
Management	1	4.73	0.030
Season	1	7.95	0.019
Habitat	1	34.62	<0.001
Host: Auchenorrhyncha	1	12.54	<0.001
Management × Habitat	1	4.87	0.027
***Ceranisus* sp.**			
Management	1	3.72	0.054
Season	2	24.93	<0.001
Habitat	1	4.66	0.031
Host: Thysanoptera	1	47.93	<0.001
***Telenomus* sp.**			
Management	1	3.47	0.063
Season	2	21.30	<0.001
Habitat	1	3.36	0.067
Management × Year	2	5.94	0.051
***Anagrus* sp.**			
Season	2	7.67	0.021
Year	2	11.23	0.003
Host: Auchenorrhyncha	1	33.13	<0.001
***Oligosita* sp.**			
Management	1	3.72	0.054
Habitat	1	13.68	<0.001
Year	2	5.30	0.071
Host: Auchenorrhyncha	1	8.44	0.004
**Leafhoppers**			
Management	1	9.31	0.002
Year	2	88.76	<0.001
Management × Season	2	6.78	0.03
**Thrips**			
Management	1	59.33	<0.001
Season	2	1182.80	<0.001
Habitat	1	4430.10	<0.001
Year	2	391.46	<0.001
Management × Habitat	1	25.77	<0.001
Management × Season	2	10.30	0.006

**Table 2:** Results of a PERMANOVA test showing the effect and variance explained by explanatory variables on the community composition of dominant parasitoid morphospecies in the vineyards, from 2016 to 2018. Main effects are listed by decreasing R^2^. Only significant effects (*p* < 0.05) are listed.

	Df	R^2^	F	*p*
Sampling season	2	0.091	6.334	0.001
Year	2	0.056	3.925	0.001
Habitat	1	0.052	7.221	0.001
Management	1	0.017	2.386	0.015
Management × Habitat	1	0.018	2.528	0.016
Residuals	77	0.552		
Total	109	1.000		

DF, degrees of freedom; F, test statistic; R^2^, variance explained.

## Data Availability

Publicly available datasets were analyzed in this study. This data can be found here: https://tamarkeasarlab.weebly.com/data-sets.html.

## Supplementary Materials

The following are available online at https://www.mdpi.com/2079-7737/10/1/7/s1, Table S1: Vineyard details and dates of field sampling, Table S2: Non-crop plant species in the vineyards, Table S3: Agrochemicals applied to the experimental plots, Figure S1: The cumulative number of parasitoid species in the grass and vine habitats.
