# Supplementary material for: Effect of Weed Management on the Parasitoid Community in Mediterranean Vineyards
Source: Biology (Basel). 2020 Dec 24;10(1):7. doi: 10.3390/biology10010007 (PMC7823956; doi:10.3390/biology10010007)
Supplement: Supplementary file 1 [file biology-10-00007-s001.pdf]

Table S1. Vineyard details and dates of field sampling.

Vineyard	Grape variety	Longitude	Latitude	Sampling dates
Bar	Cabernet Sauvignon	33.13125	35.54787	2016: April 17, Jun 5, July 18
Margaliyot-Yigal	Merlot	33.20502	35.54054	2016: April 17, Jun 5, July 18<br>2017: March 30, June 8, July 28
Malkiya 2000	Cabernet Sauvignon	33.09989	35.53012	2016: April 17, Jun 5, July 18<br>2017: March 30, June 8, July 28<br>2018: April 3, June 11, August 2
Malkiya Ordea	Cabernet Sauvignon	33.09747	35.51299	
Shiphon South	Cabernet Sauvignon	33.06485	35.76705	2017: April 27, June 14, July 31<br>2018: April 10, June 11, August 2
Shiphon North	Merlot	33.07266	35.76997	

Table S2. Non-crop plant species in the vineyards. +: present in at least one of the surveys, -: absent from all plots, D- dominant species.

Family	Species	Herbicide	Ground cover
Amaranthaceae	*Amaranthus sp.*	+	+
	Amaranthus albus	+	+
	Amaranthus blitoides	+	+
	Amaranthus blitum	-	+
	Amaranthus cruentus	+	-
	Amaranthus graecizans	+	+
	Amaranthus retroflexus	+	+
Amaryllidaceae	*Allium sp.*	+	-
	Allium pallens	+	-
Anacardiaceae	*Pistacia atlantica*	+	+
	Pistacia palaestina	-	+
Apiaceae	*Ammi majus*	D	D
	Bupleurum lancifolium	+	-
	Conium maculatum	-	+
	Daucus sp.	+	-
	Daucus carota	+	+
	Eryngium sp.	+	-
	Eryngium creticum	+	-
	Falcaria vulgaris	+	+
	Ferula sp.	-	+
	Ferula communis	+	-
	Foeniculum sp.	D	-
	Foeniculum vulgare	+	-
	Lagoecia cuminoides	+	-
	Ridolfia segetum	+	-
	Scandix pecten-veneris	+	-
	Synelcosciadium carmeli	+	-
	Tordylium sp.	+	+
	Tordylium trachycarpa	+	+
	Torilis sp.	+	+
	Torilis arvensis	+	+
	Torilis nodosa	+	+
Araceae	*Arum sp.*	+	+
	Arum palaestinum	-	+
	Eminium spiculatum	+	-
Asclepiadaceae	*Cynanchum acutum*	+	-
Asparagaceae	*Asparagus sp.*	+	+
	Asparagus aphyllus	+	+
	Bellevalia sp.	+	+
	Ornithogalum sp.	-	+
	Ornithogalum narbonense	+	-
Asteraceae	*Anthemis sp.*	+	+
	Anthemis bornmuelleri	-	+
	Calendula sp.	-	D
	Calendula arvensis	+	+
	Carduus argentatus	+	+
	Carthamus glaucus	+	+
	Carthamus tenuis	-	+
	Centaurea iberica	+	+
	Centaurea sp.	+	+
	Cichorium pumilum	+	+
	Conyza sp.	D	+
	Conyza bonariensis	+	+
	Conyza canadensis	-	+
	Crepis sp.	-	+
	Crepis aculeata	-	+
	Crepis aspera	+	+

Family	Species	Herbicide	Ground cover
	Crepis palaestina	+	+
	Crepis sancta	+	+
	Crepis syriaca	+	-
	Dittrichia viscosa	-	+
	Echinops sp.	+	+
	Hedypnois cretica	-	+
	Helminthotheca echioides	+	+
	Lactuca sp.	D	D
	Lactuca saligna	+	+
	Lactuca serriola	+	+
	Leontodon tuberosa	-	+
	Notobasis syriaca	-	+
	Onopordum sp.	-	+
	Picris altissima	+	D
	Rhagadiolus stellatus	-	+
	Senecio vernalis	-	+
	Silybum marianum	+	+
	Sonchus oleraceus	+	+
	Tolpis virgata	D	+
Asteraceae (continued)	*Tragopogon coelesyriacus*	+	+
	Tragopogon longirostris	+	+
	Urospermum picroides	+	+
	Xanthium spinosum	+	-
Boraginaceae	*Anchusa sp.*	+	-
	Echium glomeratum	+	-
	Heliotropium sp.	+	+
	Heliotropium bovei	+	+
Brassicaceae	*Capsella bursa-pastoris*	+	+
	Fibigia clypeata	+	+
	Hirschfeldia incana	-	+
	Isatis lusitanica	-	+
	Ochthodium aegyptiacum	+	+
	Rapistrum rugosum	-	+
	Sinapis alba	-	+
	Sinapis arvensis	-	+
Capparaceae	*Capparis sp.*	+	+
	Capparis spinosa	-	+
Caryophyllaceae	*Cerastium dubium*	-	+
	Minuartia sp.	-	+
	Stelaria sp.	+	+
	Stellaria media	-	+
	Stellaria pallida	-	+
Chenopodiaceae	*Chenopodium sp.*	D	-
	Chenopodium album	+	+
	Chenopodium vulvaria	+	+
	Salsola sp.	+	+
	Salsola tragus	+	+
Convolvulaceae	*Convolvulus sp.*	D	+
	Convolvulus arvensis	+	+
	Convolvulus betonicifolius	+	+
	Convolvulus palaestinus	+	-
Cucurbitaceae	*Ecballium elaterium*	-	+
Cyperaceae	*Cyperus rotundus*	+	+
Dipsaceae	*Cephalaria joppensis*	+	+
	Lomelosia palaestina	+	-
	Lomelosia prolifera	+	-
Ephedraceae	*Ephedra sp.*	D	+
	Ephedra foeminea	+	-
Euphorbiaceae	*Chrozophora tinctoria*	+	+
	Euphorbia sp.	+	+

Family	Species	Herbicide	Ground cover
	Euphorbia aleppica	+	-
	Euphorbia chamaesyce	-	+
	Euphorbia falcata	+	-
	Euphorbia hirsuta	-	+
	Euphorbia prostrata	+	+
	Euphorbia serpens	+	+
	Mercurialis annua	+	+
Fabaceae	*Alhagi graecorum*	+	-
	Astragalus hamosus	+	+
	Medicago sp.	+	+
	Medicago blancheana	+	-
	Medicago constricta	+	-
	Medicago granadensis	-	+
	Medicago monspeliaca	+	-
	Medicago orbicularis	+	+
	Medicago polymorpha	+	-
	Medicago scutellata	+	-
	Onobrychis sp.	+	+
	Onobrychis squarrosa	+	+
	Ononis sp.	+	+
	Ononis pubescens	+	+
	Ononis spinosa	+	+
	Ononis viscosa	+	+
	Prosopis sp.	-	D
	Prosopis farcta	+	+
	Scorpiurus sp.	+	+
	Scorpiurus muricatus	+	+
	Tetragonolobus palaestinus	-	+
	Trifolium sp.	+	+
	Trifolium argutum	-	+
	Trifolium glanduliferum	-	+
	Trifolium pauciflorum	+	-
	Trifolium pilulare	+	+
	Trifolium purpureum	+	+
	Trifolium spumosum	+	+
	Trigonella sp.	-	+
	Vicia sp.	+	+
	Vicia sativa	+	-
Fabaceae (continued)	*Vicia palaestina*	+	-
	Vicia peregrina	+	+
	Quercus sp.	+	+
	Quercus boissieri	+	+
	Quercus calliprinos	+	+
	Quercus ithaburensis	+	+
Fumariaceae	*Fumaria densiflora*	-	+
Geraniaceae	*Erodium sp.*	+	D
	Erodium acauale	-	+
	Erodium malacoides	+	+
	Erodium moschatum	+	+
	Geranium dissectum	+	-
	Geranium molle	+	+
	Geranium robertianum	+	+
	Geranium rotundifolium	+	+
Hypericaceae	*Hypericum triquetrifolium*	+	+
Lamiaceae	*Lamium amplexicaule*	+	+
	Moluccella laevis	+	-
Malvaceae	*Alcea sp.*	-	+
	Alcea acaulis	+	-
	Alcea dissecta	+	-
	Lavatera cretica	+	+
	Malva sp.	+	+

Family	Species	Herbicide	Ground cover
	Malva nicaeensis	+	+
	Malva parviflora	-	+
Onagraceae	*Epilobium sp.*	+	-
	Epilobium parviflorum	-	+
Orobanchaceae	*Orobanche crenata*	-	+
Papaveraceae	*Papaver umbonatum*	-	+
Plumbaginaceae	*Plumbago europaea*	+	-
Pinaceae	*Pinus spp*	+	-
Poaceae	*Alopecurus sp.*	+	-
	Avena sp.	-	+
	Avena barbata	-	+
	Avena sterilis	-	+
	Brachypodium distachyon	-	+
	Bromus sp.	+	D
	Bromus fasciculatus	+	+
	Bromus japonicus	-	+
Poaceae (continued)	*Bromus madritensis*	-	+
	Bromus scoparius	-	+
	Catapodium rigidum	-	+
	Cynodon dactylon	+	+
	Hordeum sp.	D	D
	Hordeum bulbosum	+	-
	Hordeum glaucum	+	+
	Hordeum spontaneum	+	+
	Lamarckia aurea	-	-
	Lolium perenne	+	-
	Lolium rigidum	+	+
	Setaria adhaerens	+	+
	Sorghum sp.	D	D
	Sorghum halepense	+	+
	Vulpia myuros	+	+
Polygonaceae	*Polygonum sp.*	D	D
	Polygonum arenastrum	+	+
	Polygonum equisetiforme	+	+
	Rumex sp.	-	+
	Rumex pulcher	+	+
Portulacaceae	*Portulaca oleracea*	+	+
Primulaceae	*Anagallis arvensis*	-	+
Ranunculaceae	*Adonis microcarpa*	+	-
	Anemone coronaria	+	-
	Ranunculus sp.	+	+
	Ranunculus scandicinus	-	+
Rhamnaceae	*Rhamnus alaternus L.*	-	+
	Rhamnus lycioides	+	-
Rosaceae	*Crataegus sp.*	+	-
Rubiaceae	*Galium sp.*	-	+
	Galium aparine	-	+
	Galium murale	-	+
	Rubia tenuifolia	-	+
	Sherardia arvensis	+	+
Santalaceae	*Osyris alba*	+	+
Scrophulariaceae	*Verbascum sp.*	+	-
	Veronica sp.	-	+
Smilacaceae	*Smilax aspera*	-	+
Solanaceae	*Solanum villosum*	-	+
Solanaceae (continued)	*Solanum elaeagnifolium*	-	+
Xanthorrhoeaceae	*Asphodeline lutea*	+	+
Zygophyllaceae	*Tribulus terrestris*	+	+

Table S3. Agrochemicals (fungicides (F), insecticides (I) and adjuvants (A)) applied to the experimental plots (H – herbicide plot, GC – ground cover plot).

Year	Vineyard	Plot (Treatment)	Application date		Commercial name	Purpose
2016	Bar	H	April	3	Karathane Star	F
		H	April	3	Keep	F
		GC	April	11	Keep	F
		H	April	15	Kod Kod	I
		GC	April	17	Kod Kod	I
		H	April	18	Keep	F
		H	April	18	Sheriff Super	F
		GC	April	24	BB5	A
		GC	April	26	Bayfidan	F
		GC	May	17	Sulphur	F
	Yigal	Herbicide	April	1	Keep	F
		GC+H	April	1	Sheriff Super	F
		Ground cover	April	17	Keep	F
2017	Malkiya 2000	GC+H	July	9	Tarsip	I
		Herbicide	May	9	Domak combi	F
		Ground cover	May	10	Domak combi	F
		GC+H	May	24	Durivo	I
		GC+H	May	24	Skipper	F
	Ordea	GC+H	April	16	Karathane Star	F
		GC+H	April	16	Keep	F
		GC+H	April	29	Talius	F
		GC+H	July	3	Tarsip	I
		GC+H	June	16	Heliogrofit	F
		GC+H	June	16	Karathane Star	F
		GC+H	May	12	Domark Combi	F
		GC+H	May	23	Durivo	I
		GC+H	May	23	Skipper	F
2017	Shiphon North	GC+H	April	20	Athlete	F
		GC+H	April	20	Keep	F
		GC+H	April	20	Sheriff Super	F
		GC+H	August	6	Denim	I
		GC+H	July	30	Teppeki	I
		GC+H	June	12	Karthane Star	F
		GC+H	June	12	Keep	F
		GC+H	May	3	Cordon	F
		GC+H	May	3	Vivando	F
		GC+H	May	17	Cordon	F
		GC+H	May	17	Talius	F
		GC+H	May	29	Koala	F
		GC+H	May	29	Netz	F
	Shiphon South	GC+H	April	18	Athlete	F
		GC+H	April	18	Keep	F
		GC+H	April	18	Sheriff Super	F
		GC+H	August	13	BB5	A
		GC+H	August	13	Movento	I
		GC+H	June	2	Gofrativ 80	F
		GC+H	June	11	Koala	F
		GC+H	June	11	Netz	F
		GC+H	June	29	BB5	A
		GC+H	June	29	Movento	I
		GC+H	May	3	Cordon	F
		GC+H	May	3	Vivando	F
		GC+H	May	16	Cordon	F
		GC+H	May	16	Talius	F
2018	Malkiya 2000	GC+H	April	22	Dommarc Combi	F
		GC+H	April	22	Cannon	F
		GC+H	May	6	Talius	F
		GC+H	May	21	Sheriff Super	F

Year	Vineyard	Plot (Treatment)	Application date		Commercial name	Purpose
		GC+H	May	21	Avaunt	I
		GC+H	June	3	Luna	F
		GC+H	July	9	Tarsip	I
	Ordea	GC+H	May	24	Luna	F
		GC+H	May	24	Avaunt	I
		GC+H	June	9	Sulphur	F
		GC+H	June	9	Netz	F
		GC+H	June	20	Vivando	F
		GC+H	June	20	Sulphur	F
		GC+H	Aug	23	Spartha	I
2018	Shiphon North	GC+H	April	3	Sheriff Super	F
		GC+H	April	3	Athlete	F
		GC+H	April	15	Talius	F
		GC+H	April	15	Acrobat	F
		GC+H	May	10	Luna	F
		GC+H	May	10	Keep Athlete	F
		GC+H	May	22	Keep	F
		GC+H	May	22	Karathane Star	F
		GC+H	May	29	Teppeki	I
		GC+H	May	29	Cannon	I
		GC+H	June	8	Teppeki	I
		GC+H	June	11	Sulphur	I
		GC+H	July	5	Keep Athlete	I
		GC+H	July	25	Keep	I
2018	Shiphon North	GC+H	July	25	Armada	I
	(continued)	GC+H	Aug	12	Denim	I
	Shiphon South	GC+H	April	3	Sheriff Super	F
		GC+H	April	3	Athlete	F
		GC+H	April	15	Talius	F
		GC+H	April	15	Acrobat	F
		GC+H	April	29	Vivando	F
		GC+H	April	29	Orvago	F
		GC+H	May	10	Luna	F
		GC+H	May	10	Keep Athlete	F
		GC+H	May	23	Netz	F
		GC+H	May	23	Koala	F
		GC+H	May	30	Sulphur	F
		GC+H	June	10	Talius	I
		GC+H	June	10	Keep Athlete	I
		GC+H	June	18	1+2+3	I
		GC+H	August	19	Spartha	I

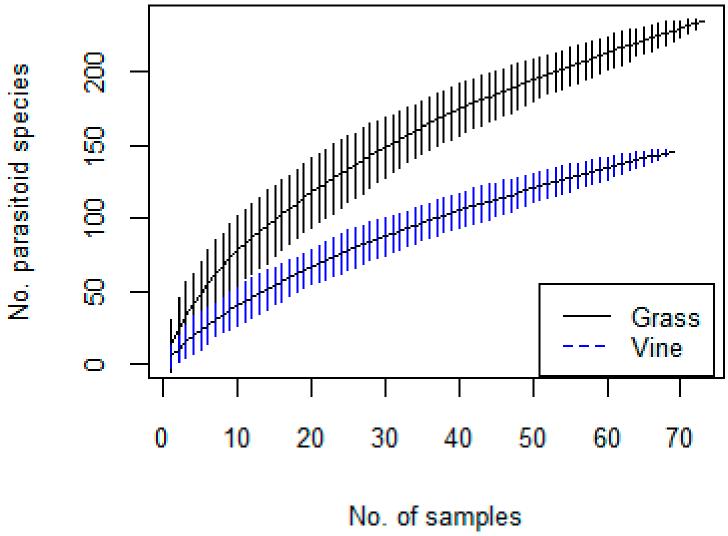

Figure S1. The cumulative number of parasitoid species in the grass and vine habitats, with associated confidence intervals.
